# The pathogenesis, risk factors, diagnosis and treatment of Acanthamoeba keratitis

**DOI:** 10.3389/fmed.2025.1559224

**Published:** 2025-07-24

**Authors:** Mingliang Bao, Hai Bao, Shuqing Wang, Hongyan Zhou

**Affiliations:** ^1^Department of Ophthalmology, China–Japan Union Hospital of Jilin University, Changchun, China; ^2^Department of Cataract, Changchun Aier Eye Hospital, Changchun, China

**Keywords:** Acanthamoeba keratitis, diagnosis, treatment, risk factors, pathogenesis

## Abstract

Acanthamoeba keratitis (AK) is a rare corneal disease that can lead to permanent visual impairment. Its incidence is relatively low when compared with that of other forms of infectious keratitis. As early clinical diagnosis of AK is challenging (e.g., overlapping symptoms, lack of specific diagnostic tools, etc.), it is often misdiagnosed as other types of infectious keratitis, such as viral keratitis or fungal keratitis. Once a patient is diagnosed with AK, the prognosis is extremely poor unless an early start of an aggressive treatment program is implemented, as timely diagnosis and treatment are closely related to a good prognosis. AK can be diagnosed through corneal scraping, culture, polymerase chain reaction, or *in vivo* confocal microscopy. Drug treatment typically involves a combination of biguanide and diamine. In advanced stages of the disease, corneal transplantation is required. This review focuses on the pathogenesis, risk factors, early diagnosis, and treatment of Acanthamoeba keratitis. This review aims to enhance the understanding of Acanthamoeba keratitis.

## 1 Introduction

Acanthamoeba, being a tiny and free-living protozoan, can generally be isolated from soil, water, air, and the nasopharyngeal mucosa of healthy individuals (1, 2). Acanthamoeba exists in two life cycle stages: trophozoites and cysts, transitioning depending on environmental conditions (3). Trophozoites, the vegetative form of Acanthamoeba, feed on organic matter and microorganisms while reproducing through mitosis (4). The trophozoites grew fastest at temperatures closer to 28°C and 37°C (5). The Acanthamoeba trophozoite is the main form of locomotion, reproduction, and infection, with a size of 17.1–58.5 μm and an average of 25.4 μm (4). It is the trophozoites that differentiate into cysts when exposed to harsh conditions such as lack of nutrients or extreme heat or cold (4). Mature cysts are round, measuring 10–25 μm, with thick double-layered walls and minimal metabolic activity (6). They are highly resistant to the external environment and can survive for up to 20 years under dry conditions (7). The ectocyst and the endocyst constitute two layers of the Acanthamoeba cyst (8). During encystment, the ectocyst forms an irregular, patchy layer composed of proteins and polysaccharides (9, 10). The endocyst, which is denser and granulated, is primarily composed of cellulose and is usually thicker than the ectocyst (9, 10). Acanthamoeba cyst walls are composed of carbohydrates (35%, mainly cellulose), proteins (33%), lipids (4–6%), ash (8%), and unknown components (20%) (9).

**Figure 1:**
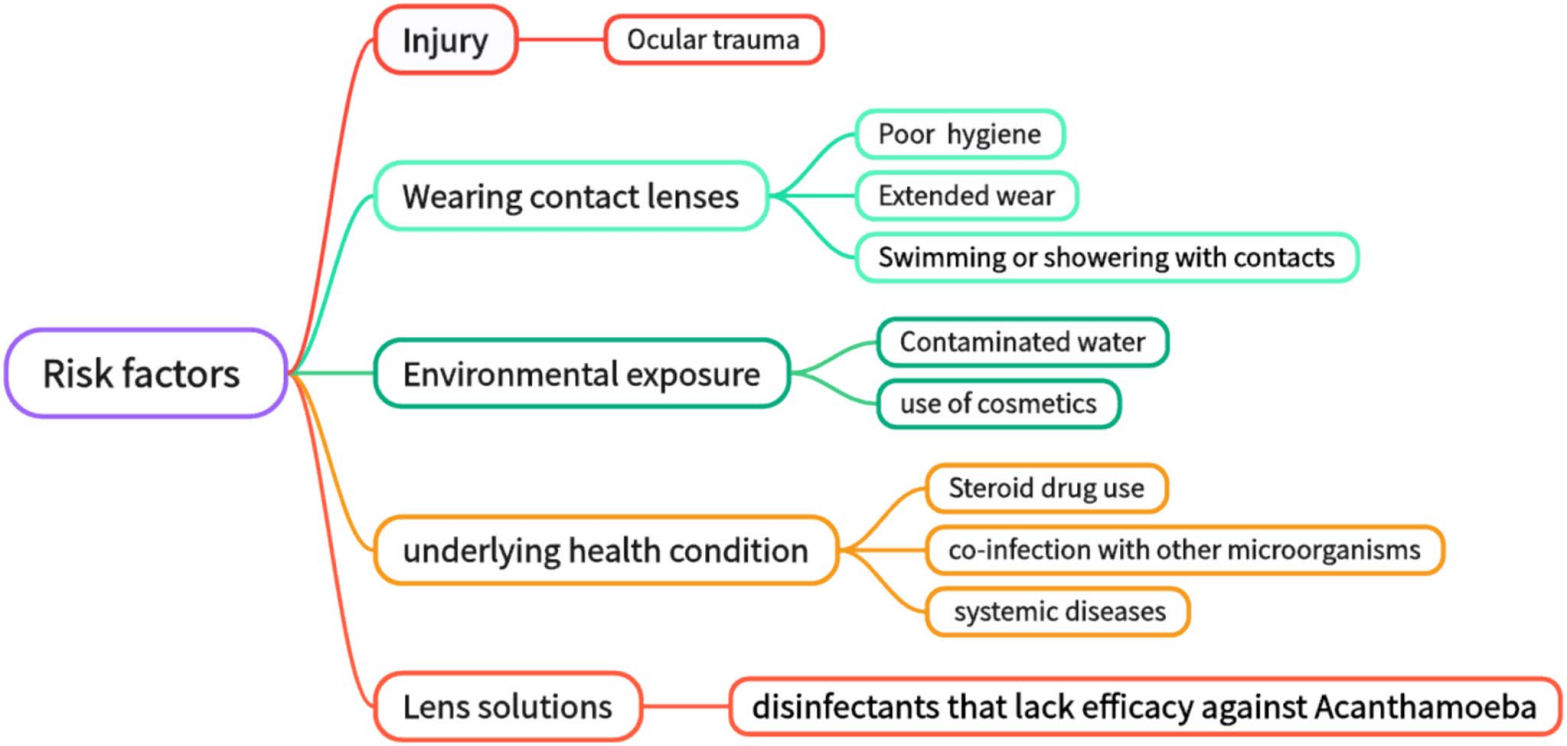
Risk factors for Acanthamoeba keratitis.

**Figure 2:**
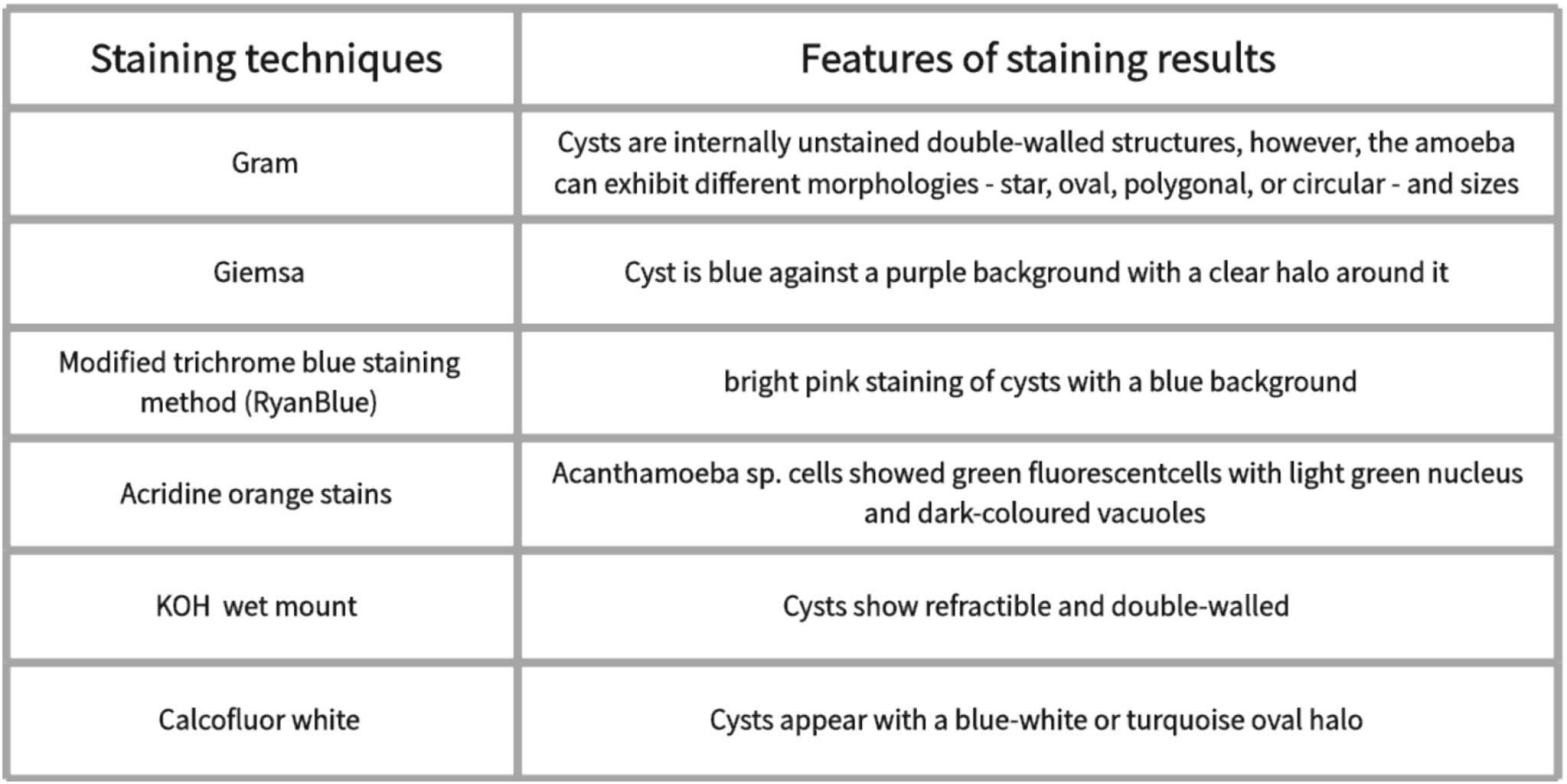
Results of staining techniques.

**Figure 3:**
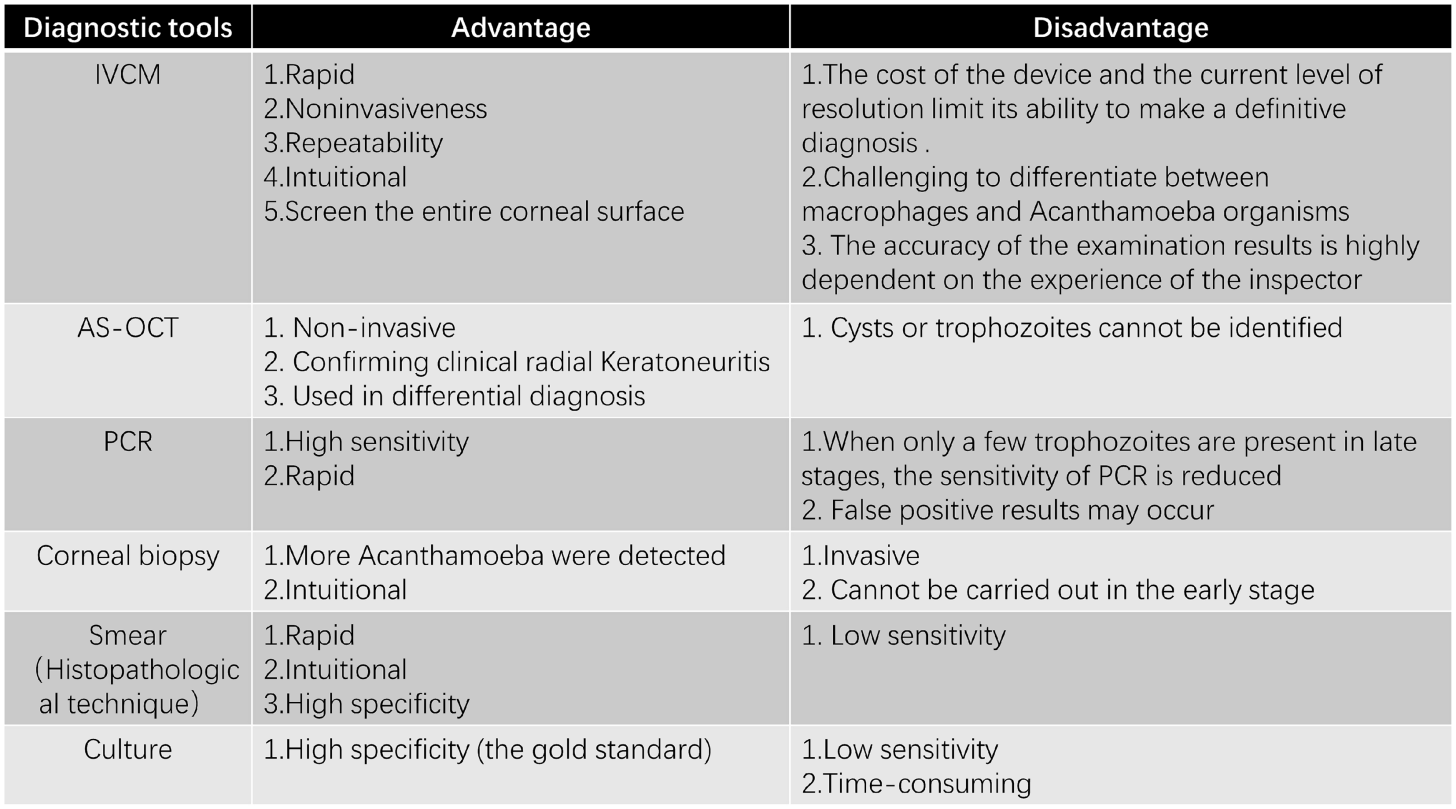
The advantages and disadvantages of various examinations.

Pathogenic Acanthamoeba, which is known to cause problems, can lead to severe infections in two separate individuals - amebic keratitis and granulomatous amebic encephalitis (GAE) (11). GAE is most commonly seen in immunocompromised patients, while Acanthamoeba keratitis occurs in immunocompetent individuals (12, 13). So far, 23 Acanthamoeba genotypes (T1 - T23) have been identified, with the identification based on the complete 18S rRNA gene sequence (14). The T4 genotype proves to be the most widespread in nature and is detected in the majority of AK-related infections (15). Most of the Acanthamoeba isolates, which were taken from the patients with the severest infections, were of the T4 genotype as well, and the T4A subgenotype in particular (16). It is T4 that is currently subdivided into eight different groups, namely T4A, T4B, T4C, T4D, T4E, T4F, T4G/T4Neff, and T4H (17). The genotyping of Acanthamoeba holds significance, for different genotypes vary in clinical manifestation and reaction to drug treatment (14). The study of Acanthamoeba genotypes and clinical correlations showed that T4C and T4D were strongly associated with better and worse outcomes, respectively (14). Acanthamoeba keratitis is a corneal illness that poses a progressive threat to vision. In recent years, with the extensive clinical application of contact lenses, the number of AK patients has been increasing year by year (18, 19). The definitive diagnosis of AK can be made by the detection of Acanthamoeba cysts or trophozoites confirmed by staining, tissue culture, or pathology. A thorough understanding of Acanthamoeba trophozoites and cysts can improve early diagnosis of AK (Figures 1–3).

## 2 Pathogenesis

The development of AK commences with the adhesion of Acanthamoeba to the surface of the cornea (20). It is the mannosylated glycoproteins on the corneal epithelium surface and the mannose receptors on the trophozoite that are the most crucial among many proteins that mediate the adhesion process (21). Then, the trophozoites overexpress the kinase to dissolve the corneal epithelium and destroy the corneal epithelial barrier through phagocytosis and apoptosis induction, resulting in severe corneal ulcer (7). Trophozoites penetrate the corneal stroma through the damaged epithelium, leading to tissue destruction (7). After entering the corneal stroma, trophozoites feed on stromal cells and decompose tissue organic particles, causing severe corneal cell injury, inducing strong corneal inflammation, and eventually causing corneal stromal necrosis (22). In addition to tissue damage caused by pathogens, the host’s immune system also plays a crucial role in the disease process (23). Acanthamoeba evades immune responses by degrading immunoglobulins and proteinase inhibitors and evading complement lysis (23–25). Acanthamoeba antigen and antibody response can be clinically manifested as corneal ring infiltration (26). The varying pathogenicity of Acanthamoeba strains causes different immune responses, which ultimately affect the progression of the disease (23). Understanding the pathogenesis and infection process of AK is very important for mastering the disease development process and timely modifying the treatment plan of AK.

## 3 Risk factors

According to statistics, the main causes of AK include ocular surface trauma and improper wearing of contact lenses (27–29). While contact lens wear is the primary risk factor for AK, some cases occur without identifiable predisposing factors (30). Ocular surface trauma is responsible for being the leading cause of the disease in developing countries (31, 32). Contact lens wearing is a major risk factor for AK in developed countries (33–35). Secondary risk factors include poor hygiene conditions, exposure to contaminated water, and improper contact lens care (swimming with contact lenses, rinsing contact lenses with non-disinfectant water or ineffective disinfectants, etc.) (36–38). In addition, environmental and systemic factors include water pollution, climate change, steroid use (which suppresses immune responses), co-infections with other microorganisms (for example, the influence of symbiotic bacteria), cosmetic use (which may introduce pathogens or damage the corneal epithelium), and complications associated with systemic disease (such as immunosuppression states like diabetes or HIV infection) (5, 36) (Figure 1). For clinically suspected AK patients, attention should be paid to the detailed inquiry of the history of ocular trauma and the history of wearing contact lenses, which has a good hint for diagnosis. Effective prevention of AK infection must be based on a thorough understanding and avoidance of AK-related risk factors. Studies indicate that contact lens wear facilitates Acanthamoeba transmission by altering the ocular surface and increasing mannosylated glycoprotein expression, enhancing trophozoite adhesion (37). The study found that 29.8% of AK patients were associated with corneal epithelial injury after wearing contact lenses (39). Studies have shown that when contact lens solutions are combined with silver nanoparticles, their anti-amoeba properties are enhanced, and the adhesion ability of Acanthamoeba to the surface of contact lenses is reduced (9, 40, 41). However, their mechanism of action is not yet fully clear. Since cellulose is present in Acanthamoeba cysts, it seems a viable idea to achieve an anti-Acanthamoeba effect by disrupting the cyst wall by adding cellulase to the contact lens solution (9, 42). However, there is currently a lack of *in vivo* experimental data.

## 4 Diagnosis

### 4.1 Clinical manifestation

#### 4.1.1 Symptom

Acanthamoeba keratitis often occurs in one eye. Contact lens wearers represent the highest-risk group for AK. This group of people may have problems such as decreased or abnormal corneal sensitivity due to long-term wearing of contact lenses, which makes them less sensitive to pain. Early symptoms of Acanthamoeba keratitis are not specific. While some patients are asymptomatic, others experience non-specific symptoms such as foreign body sensation, photophobia, tearing, or severe ocular pain (43, 44). Severe ocular pain disproportionate to physical findings is a hallmark of AK (45). However, studies report variable pain levels among patients, likely influenced by individual factors and corneal sensitivity changes due to contact lens wear (45–47). It was reported by Sun et al. (46) and Chynn et al. (45) that 55 and 91% of their patients claimed to have severe eye pain. In contrast, Sharma et al. (47) found that no patient among them had eye pain that deviated from the appropriate level corresponding to the degree of keratitis. Clinicians should be particularly cautious when severe ocular pain is disproportionate to physical findings. However, the absence of pain does not exclude AK, especially in long-term contact lens wearers with reduced corneal sensitivity.

#### 4.1.2 Physical signs

The earliest sign of AK is corneal epithelial involvement, characterized by epithelial opacities, microcysts (“dirty epithelium”), and pseudo-dendrites (37, 48). In addition, the early stage of AK corneal epithelial lesions may present with the initial appearance of suspected herpetic stromal keratitis, showing interstitial edema with intact overlying epithelium (30). In the early stage, it can also present with limbitis, perineural infiltrates, and superficial epithelial changes, occasionally accompanied by mild anterior uveitis (9). The lesion progresses further, resulting in anterior stromal involvement, followed by posterior stromal involvement, and finally lesion ring infiltration (9, 49). It was shown in a study that within Acanthamoeba keratitis instances, 100% of the cases had epithelial defects, with endothelial plaques accounting for 69.2%, radial keratoneuritis for 46.2%, and ring infiltrate for 53.8% (50). Acanthamoeba infection should be highly suggested when the above symptoms are combined. It is important to pay attention to the pathological characteristics of corneal epithelium for the early diagnosis of AK (49).

Radial keratoneuritis is considered one of the most important signs in the early diagnosis of Acanthamoeba keratitis (51). It is characterized by radial, linear, and branched corneal stroma infiltration, starting from the paracentral cornea and extending to the corneal limbus (51). The epithelium in the lesion area is often intact, and there is generally no anterior chamber reaction or a mild anterior chamber reaction (52). This sign indicates stromal invasion by Acanthamoeba. If untreated, this can progress to ring infiltration. Although radial keratoneuritis is a useful diagnostic sign, it is not always present, especially in advanced disease stages (51). The study by Bacon et al. (53) reported the incidence of radial keratoneuritis being 57% in 36 eyes diagnosed within 1 month of onset and decreasing to 29% in 24 eyes diagnosed after 2 months. Although radial keratoneuritis can occur at any stage of AK, it is more common in the early stage (51). However, it is not a specific symptom of AK; Pseudomonas keratitis has a similar presentation (54).

### 4.2 *In vivo* confocal microscopy

In vivo confocal microscopy (IVCM), which is a high-resolution imaging technique, has been utilized as a potential diagnostic means. It has been used for over 30 years to diagnose AK, offering non-invasive, real-time imaging of corneal tissue. Since the use of confocal microscopy to diagnose AK was first reported by Chew et al. (55), this rapid, non-invasive, repeatable, and intuitive technique has gradually come into use for detecting AK, especially in the early stages with atypical clinical manifestations. IVCM plays a crucial role in detecting early AK, especially when the epithelium is intact and traditional diagnostic methods may fail. Since trophozoites and cysts can reside in deeper corneal layers, they may not be detected through corneal scraping or biopsy, making IVCM especially valuable in these cases (56). In the initial phase of AK, IVCM images revealed that Acanthamoeba trophozoites and cysts were present in the corneal epithelium (52). Using IVCM to detect early AK shows that the morphological characteristics of Acanthamoeba cysts and trophozoites in each layer of the cornea are similar, as indicated by the literature (6). The diameter of Acanthamoeba cysts in IVCM is 12 to 25 microns (57, 58). IVCM showed that Acanthamoeba cysts were round and composed of a low-refractive cyst wall and a highly reflective nucleus (6). The main central structure has a regular round or irregular shape, such as a triangle, an asterisk, or a hollow ring (6). The cysts are arranged in chains or clusters; even if the cysts are very close to each other, they do not overlap with each other, and there is always a narrow space between each other (57). The diameter of Acanthamoeba trophozoites is approximately 20 to 60 μm, while the shape shows amorphous, hyperreflective, irregular wedge-like structures (59). It was in the anterior 100 μm of the corneal epithelium and the anterior stroma that Acanthamoeba cysts were mainly located (60, 61). Therefore, IVCM should focus on this area to improve the detection rate of cysts. This might account for the absence of the epithelial layer and Bowman’s layer in patients with severe AK as well as the low detection rate of cysts in advanced AK, which consequently results in difficulties in AK diagnosis (60, 61). Typical cyst images and trophozoite-like images showed 100% specificity for the diagnosis of AK, while chain or cluster cyst images showed 98.2% specificity for the diagnosis of AK (57). Typical cyst images appear as round, low-reflective structures with a highly reflective central nucleus, while trophozoite-like images are irregular and hyperreflective (57). It is important to identify trophozoites in the early assisted diagnosis of AK.

Radial keratoneuritis is one of the early signs of AK. It was IVCM that revealed highly reflective patchy lesions around radial keratoneuritis, which had never been observed in any corneal pathological condition (52, 62). IVCM shows that the sub-basal corneal nerve plexus is significantly reduced in eyes with AK, which is a finding revealed by this technique (63). The mechanism behind radial keratoneuritis may be partly due to direct Acanthamoeba attack on corneal nerves, with IVCM revealing cysts or trophozoites attached to corneal nerves in the stroma (64). IVCM has the capacity to screen the entire corneal surface, in contrast to a corneal scrape or biopsy, which only involves the examination of a small portion (65). IVCM uses a laser to penetrate the cornea’s layers, providing high-resolution, real-time images of structural and pathological changes in corneal cells, inflammatory cells, and nerves. IVCM has been successfully employed in AK patients for preoperative diagnosis, for determining the deepest location of cysts within the corneal stroma, and also for evaluating the success of postoperative phototherapeutic keratectomy (PTK) treatment (61). The main advantage of this approach is that it can be performed even when the index of suspicion for this disease is low and can be repeated for monitoring the patient’s response to treatment, which is because it is relatively non-invasive. Despite its advantages, IVCM’s high cost, existing resolution level, and reliance on operator skill may limit its use in certain clinical settings (6, 65). Additionally, its ability to differentiate between inflammatory cells and Acanthamoeba organisms remains a challenge (6).

### 4.3 Anterior segment optical coherence tomography (AS-OCT)

Anterior segment optical coherence tomography is another promising tool for early AK diagnosis (66). AS-OCT has the ability to verify clinical radial keratoneuritis as highly reflective bands whose lengths range from 20 to 200 μm and which extend obliquely within the corneal stroma (66). In infectious keratitis, AS-OCT can help differentiate pathogens by detecting endothelial plaques with clear boundaries or a gap between the plaque and the endothelium (50). These bands serve as a useful indicator for AK diagnosis (66). However, AS-OCT does not identify cysts or trophozoites of Acanthamoeba species (56, 66). In addition, one of the advantages of using AS-OCT is that it monitors disease progression and treatment response by recording corneal changes and measuring corneal thickness, providing a basis for adjusting treatment regimens (67). Unlike IVCM, which can directly visualize Acanthamoeba cysts and trophozoites, AS-OCT primarily detects structural changes in the cornea, such as radial keratoneuritis and endothelial plaques.

### 4.4 Molecular biology

Molecular testing is essential for the rapid, sensitive, and specific diagnosis of keratitis (68). Among the various techniques for the diagnosis of infectious keratitis, polymerase chain reaction (PCR), which is the most recent diagnostic technique in AK, is the most sensitive (68). Research has shown that high sensitivity and the ability to provide rapid results are the advantages of PCR over culture (69, 70). The high sensitivity and reliability of PCR are significantly related to the pretreatment process (70). The presence of trophozoites, which are abundant in the early stage of AK, contributes to the high sensitivity of PCR in early diagnosis (70, 71). PCR diagnosis of Acanthamoeba keratitis was found to be stable because PCR tests based on the 18S rDNA gene are highly specific for the genus Acanthamoeba, and different strains can be identified by short (<500 bp) 18S rDNA fragments (71, 72). By combining various PCR detection methods and enhancing sample quality, the diagnostic sensitivity can be improved (73). The minimum gDNA concentration for obtaining significant amplification was 1 pg./μl for conventional PCR and 0.1 pg./μl for real-time PCR (74). Despite its advantages, PCR has limitations, including time-consuming procedures and the need for DNA isolation (75, 76). In addition, their capacity to identify at least 10 amoebae within a sample is restricted (75, 76). During the advanced stage of AK, when cysts are in the dominant position and there are scarce trophozoites, the sensitivity of PCR used for diagnosing AK is lowered (70). PCR may still be positive when the pathogen has died, but there is residual DNA/RNA (70). Various diagnostic techniques based on PCR have been worked out (75, 76). As shown in one study, two PCR-based tests that were designed for *A. castellanii* parasites had been developed in less than 3 h, thus enabling quicker diagnosis and earlier commencement of treatment (75). Holmgaard et al. (77), who relied on NGS determination, found that a specific sequence of Acanthamoeba was present. This specific sequence boasted a specificity of 100% and a sensitivity of 88% (77). Reportedly, metagenomic next-generation sequencing (mNGS) has found its application in clinical practice, and Acanthamoeba has been detected (78). The development of antibody-based diagnostic techniques led to the finding that IPNH (inosine-uridine preferring nucleoside hydrolase), which was found to be specific to Acanthamoeba, implies that its antibodies can be used as a potential reagent for the rapid differential diagnosis of AK (79). In another study, it was found that the polyclonal peptide antibody of ACAP (adenylyl cyclase-associated protein) protein could specifically detect 6 Acanthamoeba trophozoites and cysts, thereby indicating the potential to diagnose AK (80). While PCR is the most widely used molecular tool for AK diagnosis, NGS offers higher specificity, and antibody-based methods hold promise for rapid differentiation. It is noteworthy that molecular diagnosis is crucial in the diagnosis of the disease in eyes that have been previously treated with antibiotics when the culture result is negative (70).

### 4.5 Corneal biopsy

When non-invasive diagnostic techniques such as IVCM and PCR fail to confirm the diagnosis, corneal biopsy provides a means to detect Acanthamoeba cysts at deeper stromal levels. As the disease progresses, Acanthamoeba penetrates deeper into the stroma, and corneal stroma biopsy may reveal AK cysts that cannot be detected by corneal scraping and culture (81, 82). When culture results are inconclusive or negative despite disease progression, corneal biopsy is recommended to obtain deeper stromal tissue for microbiological and histopathological analysis (83). A retrospective study by Hudson et al. reported that histopathology detected Acanthamoeba more frequently than microbial culture (84, 85). Varacalli et al. reported that histological analysis of stromal biopsy had a sensitivity of 65% for detecting AK (58). Despite its diagnostic utility, corneal biopsy is an invasive procedure with potential risks, including corneal scarring and perforation. Additionally, false negatives may occur if sampling does not capture affected tissue.

### 4.6 Smear

Microscopic examination of Acanthamoeba, which is applied in clinical practice, has emerged as a significant method for the diagnosis of AK. Direct microscopic examination of a scratched smear of the cornea is very useful for visualizing cysts. Commonly used stains include Giemsa (86), hematoxylin–eosin staining (H&E staining) (87), periodic acid–Schiff (PAS) (86, 88), and Gömöri methanamine silver (86). Giemsa staining shows the Acanthamoeba cyst is blue against a purple background with a clear halo around it (89, 90) (Figure 2). Hematoxylin–eosin staining showed the presence of double-walled cysts and nucleolus (87). Classical Gram staining reveals cysts as internal unstained double-walled structures (49). However, Acanthamoeba cysts can exhibit various morphologies, including star-shaped, oval, polygonal, or circular forms, with variable sizes (49). The fluorescent stains used for the identification of Acanthamoeba pathogens include calcofluor white (30, 81, 91) and acridine orange (92, 93). Acanthamoeba cysts can be stained blue-white by Calcofluor white staining (81, 91, 94). Acanthamoeba sp. cells showed green fluorescent cells with a light green nucleus and dark-colored vacuoles by acridine orange staining (92). Modified trichrome blue staining method (Ryan Blue) showed bright pink staining of Acanthamoeba cysts with a blue background (89). Other methods include KOH mount and impression cytology. Potassium hydroxide (KOH) wet mount reveals refractile and double-walled cysts (49). Impression cytology has proven valuable in diagnosing AK when superficial lesions are involved (95). However, if the stroma is involved, both scraping and culture may be negative (49). Giemsa, Gram, and Calcofluor white stains are simple, rapid techniques with high specificity (100%) for AK diagnosis; however, their sensitivity is relatively low (55–64%) (38). While Gram, Giemsa, and PAS stains are commonly used for their accessibility and specificity, their sensitivity remains low. Fluorescent stains such as Calcofluor white and acridine orange provide enhanced visualization of Acanthamoeba cysts but may require specialized equipment. Impression cytology can aid in diagnosing superficial infections but is less useful for deeper stromal involvement. Despite its usefulness in rapid diagnosis, smear examination has limitations, including low sensitivity, variability in staining quality, and dependence on examiner expertise.

### 4.7 Culture

The current gold standard for AK diagnosis in clinical practice remains Acanthamoeba culture. The disadvantage of conventional culture is that it needs a fairly extended incubation stage and exhibits low sensitivity (74). Prior antibiotic therapy or exposure to benzalkonium chloride may inhibit Acanthamoeba growth, potentially leading to false negative (96, 97). Despite its low sensitivity and variable positivity rate, Acanthamoeba culture remains a viable diagnostic method when IVCM and PCR are unavailable (90). The culture has 100% specificity (74). In addition, the culture of Acanthamoeba can be used for antimicrobial susceptibility tests, which can help select effective therapeutic agents (98). Culture has the value of both diagnosing and guiding treatment in AK. Non-nutrient agar (NNA) with *Escherichia coli* overlay is the most widely used culture method for isolating Acanthamoeba (99). Alternative media, such as Page’s amoeba saline, can also be used for growth and identification (100). A recent study indicated that Acanthamoeba was successfully cultured on a Sabouraud dextrose agar plate painted with heat-treated dead bacilli (90). The discovery that *Acanthamoeba* spp. can be rapidly and effectively cultivated in humic acid-coated magnetic nanocomposites and leech saliva-enriched culture media provides a promising method for AK diagnosis (101). While Acanthamoeba culture has near-perfect specificity, its sensitivity ranges between 40–70%, making it less reliable as a standalone diagnostic tool (74) (Figure 3).

## 5 Treatment

### 5.1 Medication

#### 5.1.1 Amebicides

Biguanides and diamines are commonly used anti-acanthamoeba drugs. Biguanides include chlorhexidine at a concentration of 0.02–0.2% and polyhexamethylene-biguanide (PHMB) at a concentration of 0.02–0.06% (102). Its positively charged molecular structure can attract the negatively charged components of the cell membrane surface of Acanthamoeba, destroying and increasing the permeability of the cell membrane, thus causing the death of the pathogen (22). Diamines include 0.1% propamidine isethionate and 0.1% hexamidine (103). The mechanism of the treatment of AK by diamidine is to destroy the cell membrane, denature cytoplasmic proteins, and interfere with the DNA replication and division process of Acanthamoeba (104). Biguanides and diamines are effective against both Acanthamoeba cysts and trophozoites (103, 105). Diamines also have a synergistic effect with biguanides (104). Topical treatment with biguanides is considered the first-line treatment for AK (106, 107), while diamines are typically used in combination to enhance treatment efficacy (108). Two kinds of biguanide combinations are effective when diamine is unavailable to consider (104). Biguanides have the maximum cysticidal activity and can therefore be used as monotherapy (49, 109). Lim et al. indicated that chlorhexidine was approximately 86% effective when used as monotherapy, whereas PHMB was approximately 78% effective (106). It has been demonstrated that monotherapy with PHMB 0.08% is not less effective than dual therapy, yet it is not more effective either (107). The study has shown that the effect of PHMB on Acanthamoeba may be enhanced by the ATPase inhibitor ouabain (110). Currently, it has been found that the application of propamidine isothiocyanate polyclonal antibody immunoconjugate in AK therapy shows a higher efficacy in killing Amoeba (111).

#### 5.1.2 Antifungal agents (azoles as anti-Acanthamoeba agents)

The azole family is a class of antifungal drugs that includes imidazole (e.g., miconazole, ketoconazole) and triazole (e.g., itraconazole, voriconazole, and fluconazole) (104, 112). It mainly inhibits the growth and reproduction of Acanthamoeba by inhibiting the biosynthesis of ergosterol (an important component of the cell membrane) on the cell membrane, leading to structural and functional abnormalities of the cell membrane (104). Polyene antifungal drugs include amphotericin B and natamycin (112). All of the above drugs exhibit anti-amebic activity against Acanthamoeba isolates (112). Additionally, voriconazole and natamycin exhibited cysticidal activity (112). The absence of induction of trophozoite encystment processes is a crucial feature of a drug in AK treatment, and voriconazole matches this characteristic (113). Musayeva et al. (114) pointed out that the triple-combination regimen consisting of 1% voriconazole, 0.02% PHMB, and 0.1% propamidine isethionate achieved exciting results, with all the patients responding to this regimen. There were two instances of recalcitrant keratitis that exhibited resistance to alternative treatment modalities yet achieved a full resolution subsequent to the oral administration of voriconazole (115). Furthermore, Hollhumer et al. (116) reported that the duration of anti-Acanthamoeba therapy (AAT) in patients receiving adjuvant oral voriconazole treatment was shortened from an average of 12 months to 9 months.

#### 5.1.3 Antiparasitic agents and antibiotics

As an antiparasitic drug and a novel anti-Acanthamoeba drug, mitifosine (MF) induces the apoptosis of acanthamoeba by inhibiting protease kinase B (117). A study of 15 refractory AK eyes treated with oral mitifosine as salvage therapy showed that 14 cases (93.3%) were clinically cured, and 11 cases (73.3%) developed severe inflammation, among which 10 cases were treated with corticosteroids (118). Nano-chitosan was recently discovered to serve as an ideal carrier to cut down the cytotoxicity of MF (119). Mitifosine-loaded chitosan nanoparticles (Mf-cs-nps) not only reduced the toxicity of MF but also improved its efficacy in killing amoeba (119). However, the effect of topical application of mitifosine in the treatment of AK is not satisfactoryn (120). Antibiotics such as neomycin, which inhibits protein synthesis by binding to ribosomal subunits, and polymyxin B, which acts by binding to and disrupting the microbial cell membrane, are commonly used in the treatment of Acanthamoeba keratitis (121, 122). Neomycin’s effectiveness stems from its ability to both diminish trophozoite populations and disrupt the bacterial microbiome upon which Acanthamoeba depends for metabolic sustenance (123). In addition, the study indicated that neomycin alone is ineffective unless used in combination with propionamide (112).

### 5.2 Surgery

#### 5.2.1 Therapeutic epithelial debridement (TED)

For most early AK cases with parasites confined to the epithelial layer, epithelial debridement is an effective treatment strategy (124). Epithelial debridement can directly remove pathogens and promote drug penetration into the corneal tissue. The procedure can be repeated depending on the condition (46). Blaser et al. (48) described a protocol starting with TED, followed by the use of 0.1% propamidine and 0.02% PHMB, which reported a 97.8% success rate with only one eye (2%) requiring penetrating keratoplasty (PK) out of 46 eyes. The study indicated that the protocol of starting treatment with therapeutic epithelial debridement, followed by a combination of biguanides, diamines, and antibacterial agents, is a powerful initial treatment option (125). Alcohol-assisted epithelial debridement allows the diseased epithelial layer to be removed from the corneal surface as an intact sheet, preserving the tissue structure and facilitating histopathological and ultrastructural examination (124). However, corneal detachment may impair the regenerative capacity of the corneal epithelium and increase the risk of infection spreading (126).

#### 5.2.2 Keratoplasty

If the corneal inflammatory response is not controlled after treatment with anti-amoebic drugs, therapeutic corneal transplantation should be considered. Clinical presentations include continued expansion of the corneal ulcer area and gradual increase in hypopyon (126). Penetrating keratoplasty (PKP) and deep anterior lamellar keratoplasty (DALK) are regarded as the mainstream surgical options, with the choice of the proper one depending predominantly on the depth of the corneal ulcer (126). DALK was selected when the infection did not involve the Descemet’s membrane (DM) layer, while PKP was chosen when corneal perforation occurred, corneal endothelial decompensation was evaluated before the operation, and the lesion had involved the entire layer (126). DALK has a higher graft survival rate and better visual effects (126, 127). Qi et al. (126) indicated that the graft survival rates three years after PKP and BB-DALK were 61.1 and 89.5%, respectively, showing a statistically significant difference. Tew et al. (128) and Wei-Li et al. (129) indicated that the graft transparency one year after PKP was 50% (5/10) and 78.6% (11/14), respectively. Studies have indicated that a smaller size of PKP grafts (<8.5 mm) is associated with better outcomes (128, 129). Furthermore, some studies have described that PKP can lead to complications such as corneal scar formation, anterior synechia, cataracts, and glaucoma (129, 130). AAT was continued after the operation, but glucocorticoids were not used in the early postoperative period (126). The diseased corneal tissues resected after corneal transplantation were all subjected to histopathological staining to determine whether there were cysts in the corneal tissues at the edge of the lesion. AK recurrence is more likely in cases where corticosteroids were administered before AAT or when hypopyon developed (126, 131). Infiltration of the corneal graft or bed, or worsening of the anterior chamber reaction, suggests a possible recurrence (131). IVCM, corneal scraping smear, or biopsy culture demonstrating the presence of Acanthamoeba pathogens can confirm recurrence. After PKP, Acanthamoeba recurrence often occurs at the graft-host junction, while after DALK, Acanthamoeba recurrence also occurs at the graft-recipient bed junction (126). According to literature reports, the recurrence rate of Acanthamoeba keratitis after AK keratoplasty is 9.8–41% (126, 131, 132). In studies where surgical resection margins exceeded the lesion area by 1.5 mm and 1 mm, the postoperative recurrence rates were 9.8 and 16.9%, respectively (126, 131). For optimal timing of corneal transplantation, studies in developed countries recommend a minimum of 3 months of medical therapy and performing optical keratoplasty (OKP) only once inflammation has resolved and stromal scarring is evident (133). This approach is capable of reducing the reappearance of the infection and the requirement for repeated corneal transplants (133, 134).

### 5.3 Novel therapy

#### 5.3.1 Phototherapeutic keratectomy (PTK)

Because AK is highly resistant to drug therapy, PTK may be considered when drug therapy is ineffective and the amebic lesion tends to worsen, which appears to be effective in treating AK (61). PTK is suitable for AK patients whose lesions are not advanced or whose corneal epithelium is intact. PTK, by means of thermal removal, raises the likelihood of a significant decrease in the concentration of corneal Acanthamoeba (61). The process may disrupt the cyst wall, making the cyst more susceptible to chemotherapeutic agents (61). PTK not only directly eliminates amoebic cysts and necrotic tissue but also enhances treatment (61). This outcome is achieved by the excision of the Bowman layer and anterior stromal tissue, which leads to an improvement in drug penetration (61). However, patients with advanced AK or deep stromal infection cannot benefit from this treatment.

#### 5.3.2 Photodynamic therapy (PDT)

Photodynamic therapy (PDT) has shown promise as an innovative technology capable of specifically targeting pathogens, making it a potential therapeutic application. The principle of the technique is that reactive oxygen species (ROS), which are produced by visible light or specific wavelengths of light and trigger light-sensitive compounds, can cause cell death in the target pathogen or tissue (135, 136). Compared with traditional anti-infective treatments, PDT has lower cytotoxicity (137). However, reactive oxygen species (ROS) are not specific enough and may damage surrounding healthy tissue. Current research is mostly limited to *in vitro* studies, and there is a lack of large-scale clinical trials to validate the results.

#### 5.3.3 Medicine

Recently, the anti-acanthamoeba effect of repurposed poly (ADP-ribose) polymerase inhibitor AZ9482 was found (138). Surprisingly, AZ9482 caused the death of trophozoite necrotic cells rather than apoptosis (138). Although this therapy is innovative, its mechanism of action and long-term safety (such as whether it affects host DNA repair) remain unclear. The fact that the main component of the Acanthamoeba cyst wall is cellulose makes the cellulase enzyme bring new hope for AK treatment. It has been shown that cellulase enzyme combined with chlorhexidine in Acanthamoeba cysts can effectively eradicate cyst viability because the cellulase enzyme will target the tolerant shell and then use chlorhexidine to degrade the amoeba (9, 139). However, this combination therapy still faces practical challenges, such as maintaining the activity of cellulase in the corneal environment and the possibility of enzymatic degradation products triggering inflammatory reactions and exacerbating corneal damage. Nanoparticles are combined with existing or novel drugs as a potential therapeutic option because of their small size and extensive surface area, making them ideal for drug delivery and improved efficacy (40). One study found that the copper (II) coordination compounds were effective against both forms of *A. castellanii* infection. In addition, compared with corneal epithelium, the copper (II) coordination compounds are more selective to trophozoites, have less cytotoxicity, and have a good synergistic effect when combined with chlorhexidine (140). However, the above studies lack *in vivo* experimental data, and the efficacy of AK remains uncertain.

## 6 Discussion

The key to maintaining favorable vision lies in the early diagnosis and treatment of Acanthamoeba keratitis. The initial epithelial forms, particularly pseudodendritic ones, are often misdiagnosed as herpes viral keratitis, while the advanced forms, including ring infiltration, may be mistaken for fungal keratitis (141). The early symptoms of AK, including eye pain, foreign body sensation, photophobia, and vision loss, are not specific (44). However, when several of the symptoms of corneal epitheliopathy, endothelial plaque, radial keratoneuritis, and annular infiltration are combined, Acanthamoeba infection should be highly indicated, especially in cases where conventional antibacterial or antiviral therapy has failed (50). The differences in the clinical manifestations of AK may be related to differences in virulence between different strains of Acanthamoeba, the pathogenic process, differences in host immunity, and corneal sensitivity, but the exact mechanism remains to be further studied (23). The key to clinical diagnosis of AK is identifying Acanthamoeba cysts or trophozoites. The simple and quick methods include corneal scraping microscopy and IVCM examination, which can serve as the primary method for early diagnosis (64, 89). Since Acanthamoeba cysts and trophozoites are small in size, have a low density under the microscope, and can easily be confused with inflammatory cells and corneal cells, it is particularly important to accurately recognize the morphology of Acanthamoeba, carefully search for them under the microscope, and distinguish them meticulously (6). When the results of the above examinations are negative, further diagnostic confirmation can be achieved through PCR, culture, and other tests to avoid missed diagnoses (73, 75). Epithelial stage AK tends to be more straightforward to diagnose by means of a corneal scrape. This is due to the fact that there is a greater quantity of infected tissue that can be easily reached on the corneal surface (37). In contrast, advanced AK infections, which involve deeper stromal infiltration, might necessitate a more extensive scraping or even more intrusive procedures like a corneal biopsy in order to procure a sufficient amount of infected samples (37). Therefore, the epithelial phase AK may be a crucial period of opportunity where diagnostic tests are more productive and less invasive.

Currently, the treatment of AK remains a significant challenge. Mature cysts respond significantly worse to treatment than trophozoites and immature cysts, so initial aggressive treatment is a very critical step in AK management (56, 90, 142). Cysts are extremely resistant and insensitive to a large assortment of drugs, including antibiotics, antifungal agents, and antiviral agents, whereas the opposite is true for trophozoites (143). The solidity of amebic cysts and their ability to recover from physical and chemical damage make the treatment of AK quite intractable (9). AK treatment still faces challenges such as delayed diagnosis, limited drug options, drug resistance, and drug toxicity. Research reports indicate that TED and topical ethanol (20%) are effective initial treatment options for AK (48, 124). Based on this, the study recognized the combination of biguanides and diamines as a successful first-line drug treatment (104, 108). Most studies employ a high-frequency dosing regimen at the outset, followed by gradual dose reduction based on the patient’s medical response (48, 144). Currently, the study has shown the use of protocol-delivered treatment shows substantial clinical benefits for protocol-treated patients compared to those treated with individualized treatment (delivered variably, adjusting the intensity and length of treatment to the variable clinical response for each individual patient) (144). The medical cure rate improved from 56.3 to 87.2%, and BCVA < 2/60 reduced from 40.6 to 19.1% (144). Neomycin and 1% voriconazole can both be used as adjunctive therapy (112, 114). It is common in medical practice to use a combination of drugs to enhance their effectiveness and lower the required doses, which can help reduce side effects, lower the recurrence rate, and slow down the development of drug resistance (140). A prior history of topical steroid application before diagnosis correlates with more severe AK and less favorable treatment results (145). An *in vitro* study found that dexamethasone caused Acanthamoeba cysts to excyst and accelerated trophozoite proliferation, thereby significantly increasing trophozoites in the cornea (146). The immunosuppressive attributes of steroids are linked to an elevated risk of infection (146). However, steroids can ease pain, reduce corneal vascularization, and mitigate inflammation in AK patients (147). This beneficial effect was noted only when corticosteroids were administered following the initiation of AAT for a certain duration (148). In addition, to avoid recurrence, anti-Acanthamoeba drugs ought to be continuously administered after the cessation of topical steroid use (109). The early use of steroids might be associated with inferior outcomes, whereas the employment of steroids at a later stage of the treatment process could be more advantageous, indicating that the timing of steroid administration emerges as the crucial determinant. In the early stage, when the effect of drug therapy is not good and the corneal lesion has a tendency to develop deeper (such as deeper corneal stromal infiltration, corneal endothelial spots, etc.), surgical treatment can be considered (61). DALK has demonstrated a success rate exceeding 85% (127), good BCVA, and a low recurrence rate (11%) (127, 149). However, in cases involving deep stroma involvement, this surgery has shown a lower graft survival rate (60%) and a higher recurrence rate (20%) (149). TPK is considered a salvage therapy, basically used in cases of medically refractory and advanced AK (127, 128). OPK is best suited for rehabilitation purposes, providing better long-term results for patients recovering from active AK (125).

## 7 Conclusion

Recent progress in the diagnosis and treatment of AK has brought great hope for improving the prognosis of patients and reducing the burden of this infection that threatens vision. Encourage further research and clinical trials to evaluate the effectiveness and safety of emerging diagnosis and treatment methods. Exploring promising candidate drugs with anti-amoebic potential, including anti-trophozoite, anti-cyst, and anti-encystation activities, is crucial for guiding future AK treatment. The research exploring standardized diagnosis and treatment protocols for AK is necessary for improving the prognosis of AK patients.
